# Allopurinol Use during Pregnancy - Outcome of 31 Prospectively Ascertained Cases and a Phenotype Possibly Indicative for Teratogenicity

**DOI:** 10.1371/journal.pone.0066637

**Published:** 2013-06-19

**Authors:** Maria Hoeltzenbein, Katja Stieler, Mary Panse, Evelin Wacker, Christof Schaefer

**Affiliations:** Berlin Institute for Clinical Teratology and Drug Risk Assessment in Pregnancy, Charité Universitätsmedizin Berlin, Berlin, Germany; Medical College of Wisconsin, United States of America

## Abstract

Allopurinol is a purine analogue that inhibits xanthine oxidase. It is mainly used for the treatment of hyperuricemia in patients with gout or tumor lysis syndrome. Experience with allopurinol in pregnancy is scarce. In 2011, Kozenko et al. reported on a child with multiple malformations after maternal treatment with allopurinol throughout pregnancy. Possible teratogenicity of allopurinol was proposed due to the similarity of the pattern of malformations in children with mycophenolate embryopathy. A possible common mechanism of both drugs, i.e. disruption of purine synthesis, was discussed. We report on the outcome of 31 prospectively ascertained pregnancies with allopurinol exposure at least during first trimester. Pregnancy outcomes were 2 spontaneous abortions, 2 elective terminations of pregnancy and 27 live born children. The overall rate of major malformations (3.7%) and of spontaneous abortions (cumulative incidence 11%, 95%-CI 3–40) were both within the normal range. However, there was one child with severe malformations including microphthalmia, cleft lip and palate, renal hypoplasia, low-set ears, hearing deficit, bilateral cryptorchidism, and micropenis. The striking similarity of the anomalies in this child and the case described by Kozenko et al. might be considered as a signal for teratogenicity. Thus, we would recommend caution with allopurinol treatment in the first trimester, until further data are available.

## Introduction

Allopurinol is a purin analogue that inhibits xanthine oxidase. It is mainly used for the treatment of hyperuricemia in patients with gout or tumor lysis syndrome. As these conditions are infrequent in women of childbearing age allopurinol use in pregnancy has rarely been observed. The possible antioxidant function of allopurinol has recently led to an increasing discussion on extending treatment indications to cardiovascular disease[1] and pre-eclampsia [2]. In addition, allopurinol is used as adjunct therapy in order to increase efficacy of thiopurines in inflammatory bowel disease, a condition frequently affecting women of childbearing age [3], [4].

A recent publication reported on normal pregnancy outcome after allopurinol treatment throughout pregnancy [5]. On the other hand, possible teratogenicity of allopurinol had been proposed after observation of a child with multiple malformations after maternal treatment throughout pregnancy [6]. So far, there are no prospective data on allopurinol use in pregnancy. Therefore, counseling of allopurinol is difficult. We report the first case series of prospectively followed pregnancies exposed to allopurinol.

## Materials and Methods

### Ascertainment of cases and follow-up procedure

The Berlin Institute for Clinical Teratology and Drug Risk Assessment in Pregnancy offers risk assessment to physicians, other health care providers (HCP) and pregnant women. Patient data are documented through the risk consultation. Usually, data are ascertained prospectively, i.e. neither the outcome of pregnancy nor the results of prenatal diagnostics are primarily known, but are ascertained at a later stage. In retrospectively reported cases, the outcome of pregnancy is already known and has usually prompted the initial contact to our institute. Using structured questionnaires, all relevant data with respect to medications, exposures to other agents and/or diseases and course and outcome of pregnancy are documented with informed consent of the patient. The following information is obtained: obstetric and medical history, family history, patient's profession and education, treatment indications and details of exposures (timing in pregnancy, duration, dose and concomitant medication, vitamins, in particular folic acid), assisted conception, inadvertent use of hormonal contraceptives during pregnancy, social or illicit drugs and smoking. About 8 weeks after the expected date of delivery, information about complications during pregnancy (i.e. infections, gestational diabetes, preeclampsia, etc.), details in case of pregnancy loss, gestational age at birth, sex, birth weight, length, head circumference, pH, and Apgar scores and if applicable details of congenital anomalies and postnatal disorders during neonatal period are obtained. For further details on the methodology see [7].

### Ethics

The study is based on observational data and was approved by the Ethics Committee of the Charité-Universitätsmedizin Berlin, Germany. All patients were informed that their medical information will be stored and used for future scientific research. In case they have contacted us directly they have provided us with a written consent. In case their HCP have contacted us, the HCP confirm that their patients have agreed. All our correspondences to patients and HCP contain information about our data handling and the patient's rights.

### Study design

Prospectively ascertained pregnancies with maternal allopurinol exposure were identified from 1991 until June 2012. Since the vulnerable phase for birth defects induced by teratogens is limited to the 1^st^ trimester, only pregnancies with exposure to allopurinol at least between last menstrual period (LMP) and gestational week 12 were included in the study. Cases were included independent of treatment indication and exposure interval within the 1^st^ trimester. Exposure to allopurinol may have lasted longer than week 12.

The primary goal of the study was to determine the rate of major birth defects, defined as structural abnormalities of medical, surgical or cosmetic relevance induced during embryogenesis. Special emphasis was to further evaluate the teratogenic potential of allopurinol as proposed by Kozenko et al. [6]. All birth defects were classified according to Merks [8] and Rasmussen [9]. Secondary endpoints of our study were the rates of miscarriage, stillbirth and preterm delivery (<37 weeks). Weeks of pregnancy were defined by LMP. Birth weight was adjusted to gestational age at birth and sex. Percentile categories were calculated according to Voigt et al. [10].

### Statistical analysis

The birth defect rate was calculated using live births plus pregnancy losses with pathology. For calculating rates of major birth defects, genetic or chromosomal disorders were excluded. Because of the limited number of exposed cases we did not use a control group.

Since crude rates of pregnancy outcome based on observational data are biased we calculated cumulative incidences of spontaneous abortions, elective terminations of pregnancy (ETOP) and live births. For further details of this method see Meister et al. [11].

## Results

### Maternal characteristics

**Figure 1:**
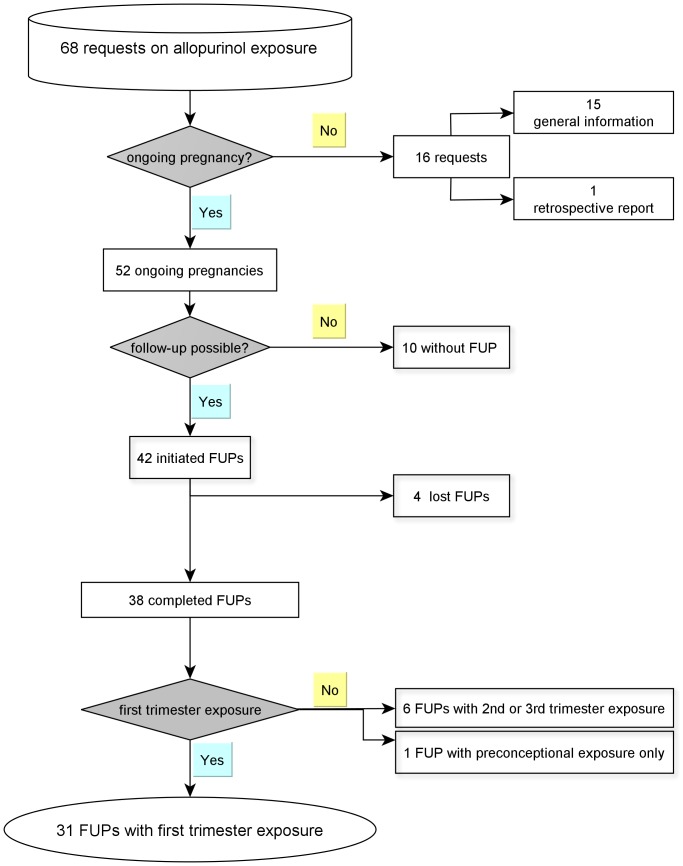
Flow chart on cases of allopurinol exposure and pregnancy (FUP =  Follow-up).

From January 1991 until June 2012 a total of 38 pregnancies with maternal allopurinol exposure and complete follow-up were identified (Fig. 1). Thirty-one pregnancies with first trimester exposure fulfilled the criteria of being prospectively ascertained and were included in this study for evaluation of pregnancy outcome. One pregnancy with uneventful outcome after long term allopurinol exposure was reported retrospectively and was therefore not considered for relative risk calculation.

Treatment indications were hyperuricemia (n = 18), gout (n = 4), renal diseases (n = 4), rheumatoid arthritis (n = 2), and systemic lupus erythematosus, chronic myeloic leucemia, hyperoxaluria (each n = 1).

**Figure 2:**
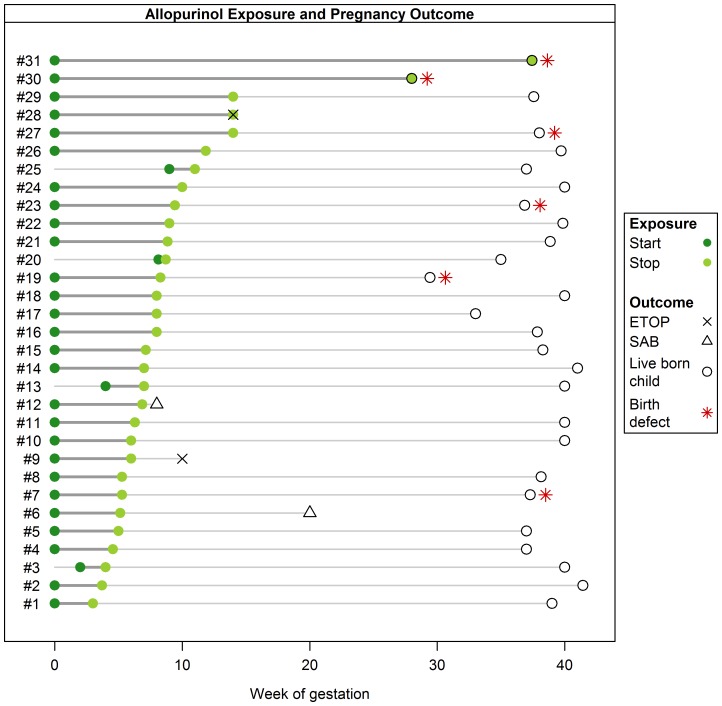
Diagram summarizing pregnancy interval of allopurinol exposure, week at first contact and outcome of 31 prospectively ascertained pregnancies.

Median dosage of allopurinol was 100 mg (range 50–600 mg/d, interquartile range (IQR) 100–300 mg/d). Treatment with allopurinol was initiated before pregnancy in 27 women. Four patients started allopurinol during 1^st^ trimester. 15/31 patients stopped therapy before week 8 after LMP, only 2 women continued treatment with allopurinol throughout pregnancy (pregnancies #30 and #31) (see Fig. 2).

Except for one mother treated with mycophenolate until week 6+6 after kidney transplantation (pregnancy #12) and one mother with cyclophosphamide (single dose at week 7+3, pregnancy #26) there were no patients with teratogenic co-medication.

On the other hand only two women were exclusively exposed to allopurinol (pregnancies #3 and #24), indicating the high co-morbidity of our patients. Twelve women were treated for hypertension, nine of whom needed two or more antihypertensives, including methyldopa (n = 5), ACE-Inhibitors or AT II receptor inhibitors (n = 7), ß-blockers (n = 7), diuretics (n = 8), monoxidine (n = 2), dihydralazine (n = 1), and clonidine (n = 1). In addition, three women used diuretics for treatment of edema. There were three women with type 2 diabetes, two women developed gestational diabetes. Interestingly, three women were on tuberculostatic therapy including pyrazinamide, well known to reduce excretion of uric acid. Further details of maternal characteristics are summarized in Table 1. Information on BMI was available from 2005 onwards. In seven of 14 patients the BMI was >35 (see also Table 1).

**Table 1:** Maternal characteristics and obstetrical history of allopurinol exposed women.

**Age (yrs)**		32 (28–37) (22–42)	n = 30/31
**BMI** 1		33.5 (23–38) (20–50)	n = 14/31
**Educational level**	9 years exam	2 (18%)	n = 11/31
	10/11 years exam	6 (55%)	
	secondary school exam	1 (9%)	
	academic study	2 (18%)	
**Smoking**	No	26 (90%)	n = 29/31
	< = 5 cig/day	1 (3%)	
	>5 cig/day	2 (7%)	
**Alcohol**	No	30 (100%)	n = 30/31
**Previous pregnancies**	0	13 (43%)	n = 30/31
	1	5 (17%)	
	2	4 (13%)	
	3 or more	8 (27%)	
**Previous parities**	0	13 (43%)	n = 30/31
	1	7 (23%)	
	2	6 (20)	
	3 or more	4 (13%)	
**Previous miscarriages**	0	26 (90%)	n = 29/31
	1	3 (10%)	
	2 or more	0 (0%)	
**Previous children with birth defect**	0	27 (93%)	n = 29/31
	1	2 (7%)	
	2 or more	0 (0%)	
**Week at first TIS contact**		8.9 (6.9–12.8) (3.1–20)	n = 31/31

For age, BMI, and week at first TIS contact, median, interquartile range, and min/max are presented.

1 BMI was only available for cases ascertained after 2004.

First contacts to our institute were initiated via gynaecologists (n = 17), clinical geneticists (n = 6), other physicians (n = 4) and patients (n = 4).

### Pregnancy outcome

Of the 31 pregnancies exposed to allopurinol, there were 2 spontaneous abortions, 2 elective terminations of pregnancy and 27 live births (see Fig. 2).

One spontaneous abortion (pregnancy #12) occurred in a patient after renal transplantation receiving several co-medications including mycophenolate, ciclosporine and various antihypertensives. Another spontaneous abortion occurred at week 20 in a patient with hypertension (pregnancy #6) treated with valsartan and hydrochlorothiazide. Chorioamnionitis was diagnosed. No malformations were found in the fetus. Two pregnancies were terminated, both for personal reasons (pregnancy #9 and #28, see Fig. 2).

**Figure 3:**
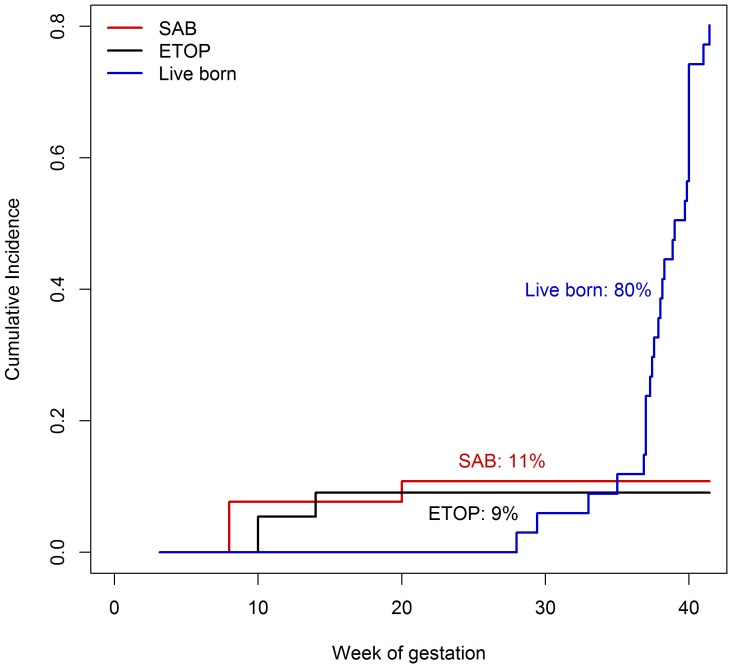
Estimation of cumulative incidences using survival analysis technique. Probability of spontaneous abortion was 11% (95%-confidence interval (CI) 3–40), ETOP 9% (95%-CI 2–32), and live birth 80% (95%-CI 60–85).

Cumulative incidences were 11% (95%-CI 3–40) for spontaneous abortions and 9% (95%-CI 2–32) for ETOPs (Fig. 3).

**Figure 4:**
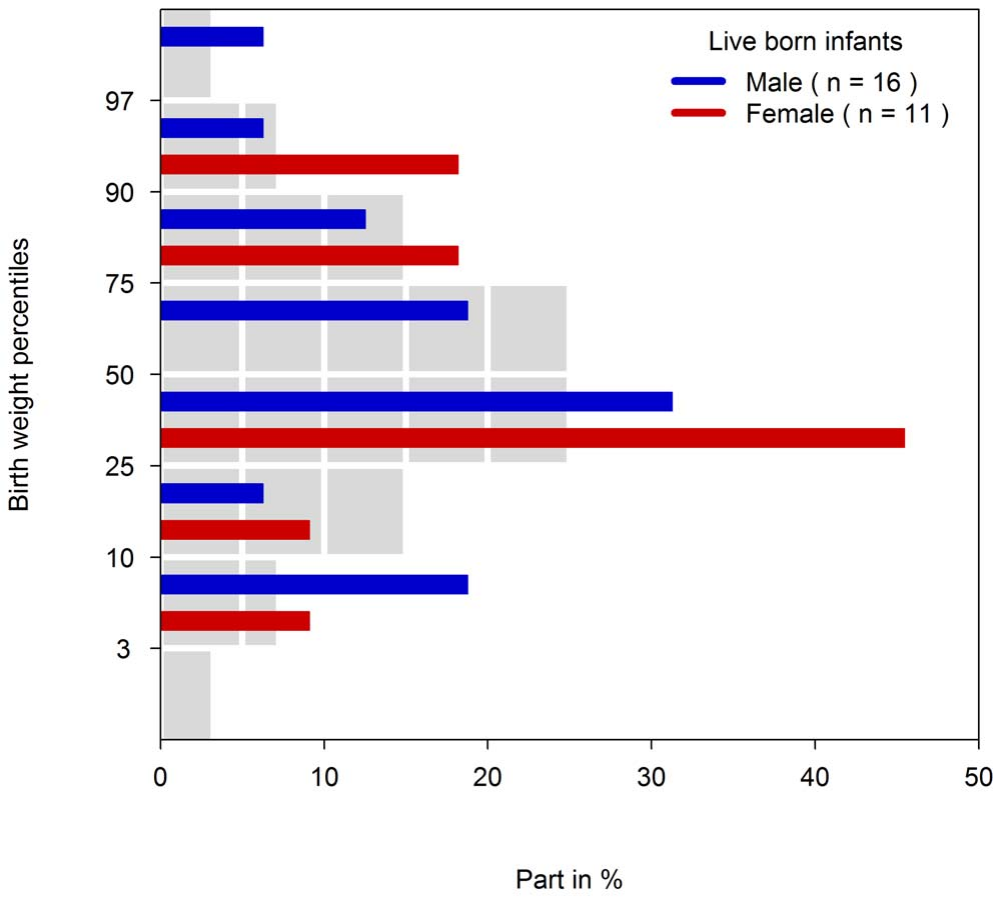
Birth weight percentiles. Bars in colors give the proportions of newborns by percentiles. Grey bars represent the proportion of newborn in the German Perinatal Project general population [10] in each percentile category.

The cumulative incidence for live births was 80% (95%-CI 60–94) (Fig. 3). There were 16 males and 11 females born alive. Five children (19%) were born premature (gestational age at birth <37 weeks). Caesarean section was performed in nearly half of the live births (13/27). Birth weights were within normal range after correction for sex and gestational age (see Table 2 and Fig. 4). Pre-eclampsia had been diagnosed in five pregnancies all exposed during 1^st^ trimester until latest week 14 and a HELLP-syndrome in one exposed throughout pregnancy (#30).

**Table 2:** Child characteristics.

**Gestational week at birth**	**38.1** (37–40) (28–41.4)	n = 27/27
**Preterm birth**	**5** (19%)	n = 27/27
**Weight** (g)	**3340** (2850–3628) (990–4690)	n = 27/27
**Length** (cm)	**50** (48–52) (36–55)	n = 26/27
**Head circumference** (cm)	**35** (33.5–36) (26–38)	n = 21/27

For gestational week at birth, weight, length and head circumference, median, interquartile range, and min/max are presented.

There were 6 infants (5 boys and 1 girl) with congenital anomalies (Table 3). Minor malformations were diagnosed in four (#7, #19, #27, #30). In patient #23 congenital hypoparathyroidism was diagnosed after birth, an autosomal-dominant condition already known in the mother.

**Table 3:** Summary of congenital anomalies after first trimester exposure with allopurinol.

Nr.	Gestational age at call (weeks)	Allopurinol exposure (weeks after LMP) and dose (mg/d)	Treatment indication	Comedication	Gestational age at birth	Congenital anomalies	Classification of anomalies
**#7**	5+2	0–5+2 (100)	Hyperuricemia	Ramipril, metoprolol, alpha-methyldopa, hydrochlorothiazide, amlodipine, simvastatin, metformin, venlafaxin, tilidine, naloxone, paracetamol, acetylsalicylic acid, insulin, insulin lispro	37+2	Patent foramen ovale, pulmonary artery stenosis (hemodynamically not relevant)	Minor
**#19**	11+3	0–8+2 (100)	Glomerulonephritis	Amlodipine, valsartan, metoprolol, hydrochlorothiazide, alpha-methyldopa, desloratadin,	29+2	Small patent ductus arteriosus, mild ptosis right eye, umbilical hernia	Minor
**#23**	9+3	0–9+3 (50)	Hyperuricemia	Calcitriol	36+6	Congenital hypoparathyroidism (autosomal-dominant)	Genetic
**#27**	15	0–14 (300)	Gout	Furosemide, pravastatin, cerivastatin	38	Hemangioma	Minor
**#30**	12+4	0–28 (100)	Hemolytic uremic syndrome	Alpha-methyldopa, metoprolol, moxonidine, furosemide clonidine, darbepoetin alfa, danaparoid, enoxaparine, corticosteroids, colecalciferol, alfacalcidol,	28	Persistent ductus arteriosus, patent foramen ovale, umbilical hernia	Minor
**#31**	5	0–37+3 (100)	Hyperoxaluria type I	Hydrochlorothiazide, sodiumcarbonate, pyridoxine	37+3	Multiple malformations (see also Table 4)	Major

One child (#31) with allopurinol exposure throughout pregnancy had multiple malformations (further details are given in Tables 3 and 4). The mother had hyperoxaluria type 1, a rare metabolic disease, and was concomitantly treated with hydrochlorothiazide (12,5 mg/d), pyridoxine (100 mg/d) and sodium carbonate.

**Table 4:** Clinical features of the patient described by Kozenko et al. [6] and our patient # 31.

	Patient from Kozenko et al.	Patient #31 from our case series
Gestational age of birth	41 weeks	37+3 weeks
Allopurinol exposure throughout pregnancy	300 mg/d	100 mg/d
Orofacial anomalies	Cleft lip and palate (right), unilateral microtia, EACA^1^, Micrognathia	Cleft lip and palate (left), low-set ears, conductive deafness, retrognathia
Ophthalmological anomalies	Microphthalmia, optic nerve hypoplasia, coloboma upper eyelid	Microphthalmia
Gastrointestinal anomalies	Diaphragmatic hernia Pulmonary agenesis (left)	Hepatosplenomegaly/cholestasis
Urogenital anomalies	Unilateral renal agenesis, bilateral cryptorchidism	Renal hypoplasia, bilateral cryptorchidism, micropenis
CNS	Hydrocephaly, hypoplasia of corpus callosum	Enlargement of ventricles
Further anomalies		Osteopenia
Cardiovascular defects	-	-
Karyotype	46,XY	46,XY

1 External auditory canal atresia.

The rate of major malformations in our cohort was 3.7% (1/27).

## Discussion

Gout, hyperuricemia and other treatment indications for allopurinol are rare in women of reproductive age. Gout usually improves in pregnancy and attacks recommence after birth, due to the decreased estrogen levels [12]. During 2^nd^ and 3^rd^ trimester allopurinol has been described for tumor lysis syndrome [13]. Gülmezoğlu et al. [14] reported its safe use in a trial including 27 women in the 3^rd^ trimester to evaluate the antioxidant potency of allopurinol for the prevention of pre-eclampsia.

To our knowledge, first trimester exposure of allopurinol has only been reported in a few case reports including 4 normal outcomes [15], [16] and a recent report [6] on a child with multiple malformations including microphthalmia, cleft lip and palate, microtia and diaphragmatic hernia after maternal allopurinol exposure throughout pregnancy (summary of features in Table 4). The authors proposed a possible teratogenic effect and noted the similarities to the mycophenolate embryopathy based on similar pathways, i.e. the interruption of purine biosynthesis. Data from animal studies indicate teratogenic effects (facial clefts and minor skeletal defects) of allopurinol at high doses in mice [17], but not in rats and other rodents [18].

We present the first prospective case series covering 31 pregnancies with 1^st^ trimester allopurinol exposure.

The rate of spontaneous abortions in our case series was in the expected range of 13–21% [19] as was the rate of major malformations (3.7%) based on one infant with multiple malformations. The infant's phenotype resembles the case report of Kozenko et al. [6]. In our infant a Fraser syndrome had been suspected initially but was not confirmed. The karyotype was normal, but further genetic analysis like array-CGH was declined by the parents. Therefore, we cannot exclude a genetic origin of this rare malformation syndrome.

The mother of our patient had hyperoxaluria, a severe renal disease. In addition to allopurinol, she was treated with hydrochlorothiazide and pyridoxine, both drugs not considered as teratogens based on animal studies and human data [20]. Pyridoxine is widely used for the treatment of hyperemesis in early pregnancy [21].

The mother of the patient reported by Kozenko et al. [6] received multivitamins and methyldopa, the latter being the first line therapy for hypertension in pregnancy and not suspected as a teratogen. Thus, our patient and the patient reported by Kozenko were not exposed to any teratogenic co-medication in pregnancy that might explain the observed malformations. Furthermore, both co-medications are not directly involved in purine metabolism.

Our case series represents women with either multiple risk factors or severe illness, known to be associated with an increased risk of poor pregnancy outcomes. The high rate of nearly 20% prematurity in our case series may be explained by maternal co-morbidity. To our surprise we did not observe lower birth weights after adjustment for sex and gestational age, which might have been expected in cases of hypertension or autoimmune diseases in pregnancy. The high number of obese women might have compensated this effect, as new-borns of obese mothers tend to be heavier than new-borns of normal weight women [22].

Obesity and hypertension were present in the majority of our women. These conditions are risk factors for hyperuricemia or gout in premenopausal women [23]. So far, no substantial malformation risk has been ascribed to the underlying maternal diseases in our cohort such as hypertension, systemic lupus erythematosus or other autoimmune diseases [24] [25]. In contrast to other metabolic diseases, such as phenylketonuria, hyperoxaluria has not been suspected to cause teratogenic effects. There are several reports on normal pregnancy outcomes in women with hyperoxaluria [26]. However, a high BMI is associated with an increased malformation risk [27], [28].

Although being the largest case series so far, our study is still limited by the small sample size. With only 2 women exposed in the 2^nd^ and 3^rd^ trimester, we are not able to examine any effect on pregnancy outcome of allopurinol exposure after the first trimester. The surprisingly high rate of 6/31 patients with pre-eclampsia or HELLP syndrome in our cohort may be explained by the underlying conditions of the women, A protective effect of allopurinol on pre-eclampsia as postulated by Gülmezoğlu et al. [14] cannot be confirmed by our data.

The important strength and advantage of our study is the prospective character of data ascertainment, allowing relative risk calculations for adverse pregnancy outcomces.

The similarity of rare malformations in the infants reported by Kozenko [6] and our study (case #31) requires further exploration by case-control studies to confirm or reject the hypothesis of allopurinol teratogenicity [29] [30].

### Conclusion

Allopurinol has been used for over 40 years without being suspected for teratogenicity in humans. This is corroborated by the overall malformation rate in our case series lying in the expected range. However, the presence of two infants with a similar rare combination of malformations including microphthalmia, cleft lip and palate, and microtia after first trimester exposure to allopurinol is striking and might be considered as a signal for teratogenicity [29]. Thus, we would recommend caution with allopurinol in the first trimester, unless further data are available. In case of inadvertent exposure during 1^st^ trimester high resolution ultrasound is recommended to confirm normal fetal development.
